# Supplementary material for: Association between oral microbiome and seven types of cancers in East Asian population: a two-sample Mendelian randomization analysis
Source: Front Mol Biosci. 2023 Nov 21;10:1327893. doi: 10.3389/fmolb.2023.1327893 (PMC10702768; doi:10.3389/fmolb.2023.1327893)
Supplement: Supplementary file 1 [file DataSheet1.ZIP › Supplementary Materials/MR plots for tongue/tongue═╝/Ovarian cancer/pheno.2962_to_ovarian cancer_forest.pdf]

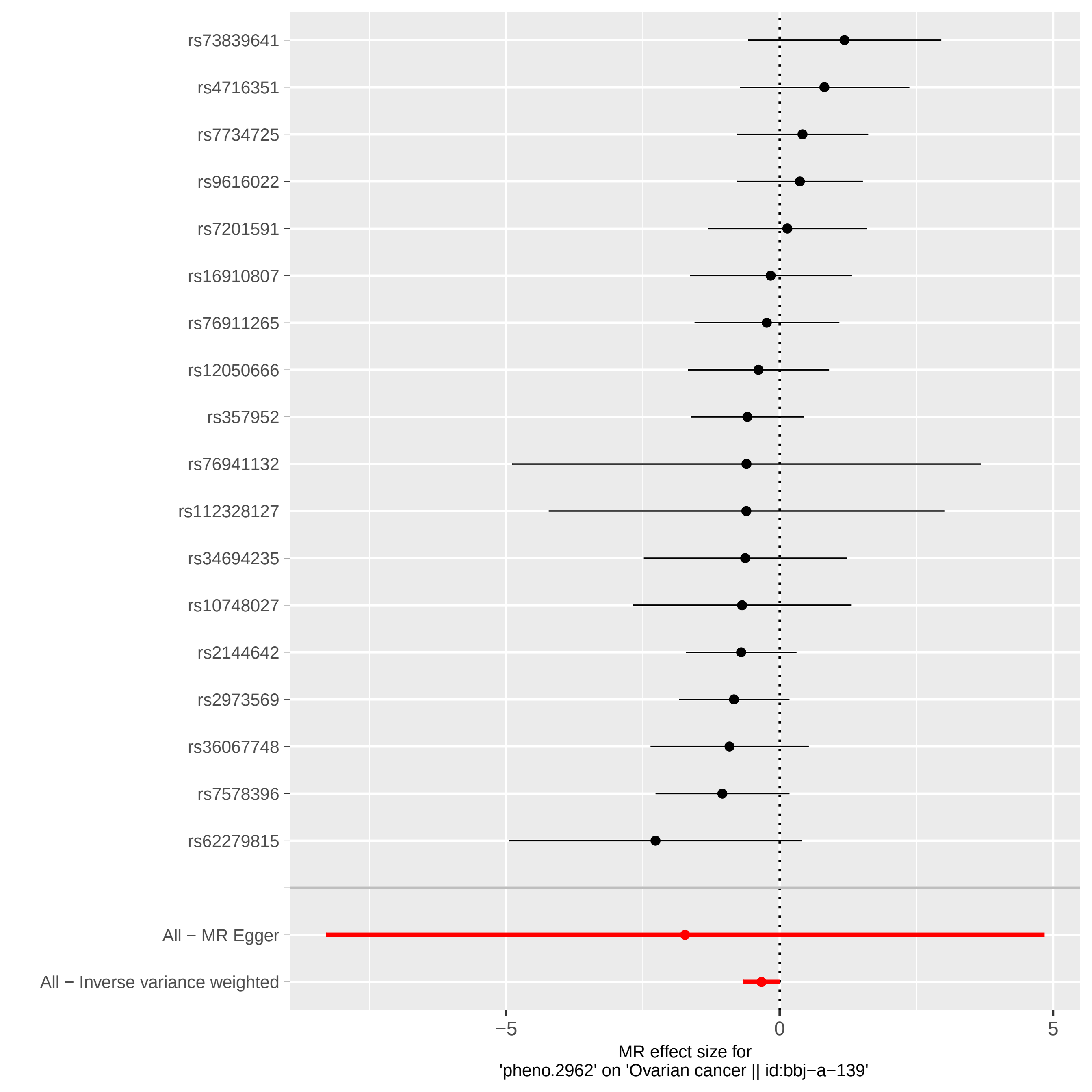
rs73839641
rs4716351
rs7734725
rs9616022
rs7201591
rs16910807
rs76911265
rs12050666
rs357952
rs76941132
rs112328127
rs34694235
rs10748027
rs2144642
rs2973569
rs36067748
rs7578396
rs62279815
All − MR Egger
All − Inverse variance weighted
−5
0
5
MR effect size for
'pheno.2962' on 'Ovarian cancer || id:bbj−a−139'
